# Unmet Supportive Cancer Care Needs: An Exploratory Quantitative Study in Rural Australia

**DOI:** 10.14740/wjon928w

**Published:** 2015-08-27

**Authors:** Krishna Rachakonda, Mathew George, Mohsen Shafiei, Christopher Oldmeadow

**Affiliations:** a Mildura Base Hospital, Mildura, VIC, Australia; b Department of Medical Oncology, Northwest Regional Cancer Centre, Tamworth, Australia; c IT and Statistical Support Unit, University of Newcastle, Hunter Medical Research Institute, NSW, Australia

**Keywords:** Palliative cancer care, Unmet needs, Supportive care, Questionnaire, Rural Australia

## Abstract

**Background:**

There is a discernible, often ignored under-evaluated care-management gap in supportive cancer care, where the estimated clinical outcome is seldom translated into patient-centered benefit.

**Methods:**

The present research is an exploratory cross-sectional quantitative questionnaire survey study done in rural regions of Australia with the sole purpose of evaluating the care-management gap in terms of the unmet supportive needs of advanced cancer patients to provide baseline data for planning, drafting and implementing innovative and effective supportive care services that will address the specific priorities and unmet needs identified in this vulnerable population in the remote and rural regions.

**Results:**

The questionnaire (NA-ACP) was comprised of 132 questions covering seven domains of supportive care. Three centers in rural regions of Australia were selected for the study. While center 1 had medical and surgical specialties, centers 2 and 3 were outreach oncology clinics with nurse-led chemotherapy units. A total sample of 75 patients getting continuous treatment procedures at these three oncology units was given the NA-ACP questionnaire.

**Conclusion:**

The data from this study can be used to improve and inform care for this population by identifying specific unmet supportive needs.

## Introduction

Patient-centered care is the described goal of healthcare today [1]. This is most relevant in palliative care settings. It is not just the advanced technology and treatment options but the cordiality expressed by a caring healthcare professional that crafts the difference in the quality of care in such settings [2, 3]. Proper care means that many terminally ill cancer patients suffer only when they do not receive adequate supportive care for the symptoms accompanying their illness. Recent research studies indicate that there are numerous unmet needs amongst cancer patients in palliative settings concerning diagnosis, disclosures, imparting and sharing of information regarding their terminal illness and providing psychological support [4]. In spite of the increasing use of surrogate end points like progression free survival, predictive biomarkers in clinical trials and introduction of newer therapies, it is critical to reveal that scores of patients abandon chemotherapy prematurely on account of the physical and psychosocial symptoms accompanying their illness [2].

Although it has been possible to classify these unmet needs as relating to activities of daily living, economics, information, physical, psychosocial and psychological aspects, systematizing specific unmet needs based on cancer type or stage has been not been possible due to heterogeneous nature of the diagnosis and identification of the needs employed in various previously published studies [5, 6]. Absence of a validated multi-level modeling tool to predict these unmet needs in various stages and types of the disease and the relatively small number of published studies available makes it difficult to generalize and synthesize the results of these studies [7].

For instance, of 94 published studies [7-17] including one RCT, one cohort study, 41 surveys, 14 structured interviews, 24 qualitative interviews and 13 focus groups, there is only one study by Mor et al [15] that prospectively measures unmet needs over a period, acknowledging the potential of unmet needs to alter the duration and course of oncogenesis. Although the first Australian study to measure unmet needs using the cancer needs questionnaire (CNQ) among participants with various cancers has been accomplished in 1995 by Foot and Sanson-Fisher [11], Lintz et al [12] have used this same classification for assessing the unmet needs of men with varied stages of disease using the supportive care needs survey [7].

Supportive care needs related specifically to physical functioning; activities of daily living and psychological support have been well elucidated by Vadivelu [18]. A question prompt list (QPL), a structured list of questions designed to encourage cancer patients to acquire information on treatment information, options and support, has been reported by McJannett et al [19]. Hohenadel et al [20] have identified six categories of unmet supportive care needs related to daily living, information, and emotional support, interaction with the healthcare team, physical health and social relationships in Canada. More recently, unmet needs have been assessed by a 34-item supportive care needs survey (SCNS-SF34). Moderate to high prevalence unmet needs in the survey pertain to psychological needs, sexuality and daily activity [21]. Eight studies look specifically at people with cancer in the palliative stages of their disease. Although six studies highlight the challenges of advanced disease on everyday activities, only one study by Aranda et al [22] reports the needs on the basis of specific cancer type, breast cancer. More than 100 unmet need categories or themes have been arrived at based on these studies [2, 7, 23, 24].

Thus, there is a discernible, often ignored under-evaluated care-management gap in palliative cancer care, where the estimated clinical outcome is seldom translated into a patient-centered benefit. These supportive care needs that call for immediate attention are classified as “unmet needs”. The unmet needs no doubt have a distressing effect on patients’ well-being [25, 26]. Supportive care needs relate not only to coping with the physical, physiological and psychological aspects of the disease during the diagnosis, disclosure and treatment, but also practical care-management issues like assistance with transportation, regulation of patient activities within the home, providing of wigs and prostheses. A scheduled periodic assessment of the cancer patients in the palliative setting is a means to swiftly identify these issues for appropriate intervention. This includes utilization of assessment tools, inventories, communication, innovation, health team co-ordination and management of resources [4, 6, 7, 17, 27].

The present research is a quantitative study done at three regional and remote areas in Australia with the sole purpose of evaluating the care-management gap in terms of the unmet supportive needs of advanced cancer patients in rural Australia to provide baseline data for planning, drafting and implementing innovative and effective supportive care services that will address the specific priorities and unmet needs identified in this vulnerable population in the remote and rural regions.

In spite of all this, there is little coherence between the numbers of unmet needs amid several physical, psychological and psychosocial need aspects reported in the literature and the mean number of needs that can be deduced to bring out a meaningful inference. At the same time, most of these studies point out to a high prevalence [4, 13, 28] stressing the need for a standardized protocol that would include all the hitherto identified unmet needs in all the domains classified, a standard methodology that would include a clearly defined time frame at various stages of the disease and types of the disease for future studies.

## Methodology

### Study objective

The primary objective of the study has been to evaluate and determine the unmet supportive care needs of advanced palliative cancer care patients as perceived by the patients in rural Australia for creating comprehensive baseline data for planning, drafting and implementing innovative and effective supportive care services.

### Study design

The present study was an exploratory cross-sectional research study comprising a questionnaire survey, data collection and analysis of the data obtained to draw definite conclusions. The questionnaire (NA-ACP) was comprised of 132 questions [5], covering seven domains of supportive care elucidated in the published literature including symptom analysis, psychological care, activities of daily living, social care, medical information, spiritual and financial care.

The domain “symptom analysis” addressed care issues including “dealing with feeling unwell a lot of the time”, “dealing with loss of appetite”, “dealing with changes to routine life style”, “dealing with working around the house” and “dealing with lack of energy and tiredness”. The domain “psychological care” addressed issues including “working through the feelings of death and dying”, “coping with the fear of losing independence”, “coping with the fear of cancer progression”, “coping with the fear of the family worries and concerns” and “coping with the frustration of not being able to do the activities done prior to diagnosis”.

The domain “activities of daily living” addressed care issues including “getting assistance with showering”, “getting assistance with walking”, “dealing with frustration of not being able to drive a car”, “getting assistance going up and down the staircase” and “getting assistance to prepare a meal”. The domain “social care” addressed issues including “coping with talking with family and friends about cancer”, “dealing with maintaining relationships with friends”, “being able to express feelings with family and friends”, “dealing with maintaining relationships with family members” and “receiving emotional support from family and friends”.

The domain “medical information” addressed care issues including “getting information about support of self, partner, family and friends”, “being able to have open discussions with the doctors”, “to be fully informed about medical test results at the earliest”, “getting information about factors that would influence the course of the disease” and “getting information about non-conventional treatment options”.

The domain “spiritual care” addressed care issues including “dealing with death and dying”, “accepting a relationship with God or a higher being”, “dealing with unmet life goals”, “setting new priorities” and “being able to choose the place where one wants to die”. The domain “financial care” addressed care issues including “availability of transport to medical appointments, shopping and work”, “obtaining sufficient financial support”, “dealing with concerns about financial situations”, “meeting basic living expenses” and “paying the non-medical cost of the illness”.

The items within each domain were rated on a numerical scale from 1 to 5, where numerical values 1 to 2 denoted “no need for help”, and 3 to 5 denoted “need for help” from low, moderate and high on the ascending order. The validated questionnaire (NA-ACP) was provided to patients undergoing treatment in advanced stages of cancer in the three rural centres selected at the time of consultation with their physician after a comprehensive explanation of the purpose of the study. A reply paid envelope was provided with a request to return the questionnaire within a week.

### Study location

Three centers in rural regions of Australia were selected for the study. Rural regions in Australia here denote the geographic area that encompasses all population, housing, and territory outside cities and towns. While center 1 (population of approximately 40,000 and 250 km from a major Metropolitan city) has medical and surgical specialties, the centers 2 (population of approximately 9,000 and a further 250 km more remote from a major Metropolitan city and center 1) and 3 (population of approximately 11,000 and 130 km from a major Metropolitan city) are outreach oncology clinics with nurse-led chemotherapy units.

### Participants

A total sample of 75 patients getting continuous treatment procedures at these three oncology units was given the NA-ACP questionnaire. Ethics committee approval and informed consent was obtained prior.

### Statistical analysis

NA-ACP questionnaire utilized by Rainbird et al has been found effective to investigate the specific needs of patients with advanced incurable cancer. The questionnaire is best suited to describe the prevalence of perceived needs among this specific population with demonstrable validity and reliability. Patient responses to the Likert scale question on unmet needs items on the overall NA-ACP questionnaire [5] were standardized for analysis (referred to here as standardized Likert scale (SLS) scores).

Data were presented in the form of means and standard deviations for continuous values or frequencies and percentage values for categorical values, with 95% confidence intervals [29]. Numbers of unmet needs as measured by the SLS score were compared between cancer types using ANOVA, and *post hoc* comparisons between cancer types were also undertaken. Statistical analysis was accomplished using SAS version 9.4 (SAS Institute, Cary, NC, USA).

## Results

### Demographics

Of the sample palliative care population of 75, there were a total of 43 male and 32 female patients. There was one patient in the 25 - 35 age group, three in the 36 - 45, eight in the 46 - 55, 14 in the 56 - 65, 25 in the 66 - 75, 22 in the 76 - 85 and two in the 86 - 90 age group. With regard to the type of cancer, there were 11 breast cancer, 23 colorectal and bowel cancer, 17 lung cancer, seven prostate cancer, five uterine, ovarian and cervical cancer, and 12 melanoma, leukaemia and lymphoma cases including non-Hodgkin’s disease. Sixty were currently receiving active chemotherapy and 15 were not receiving chemotherapy but had received it previously. Only four of them were receiving treatment with curative intent. The demographic characteristics of age, gender, cancer type, stages and treatment status are shown in Tables 1-3. The patients in the study sample were in the age group from 27 to 89 years (mean = 68). Fifty-seven percent of the sample taken was male and 43 were female.

**Table 1:** Sample Demographics

Variable	Class	Total
Gender	Male	43 (57%)
	Female	32 (43%)
Age	N	75
	Mean (SD)	68 (12)
	≤ 55	12 (16%)
	56 - 65	14 (19%)
	66 - 75	25 (33%)
	≥ 76	24 (32%)
Cancer type	Breast	11 (15%)
	Gastrointestinal	23 (31%)
	Lung	17 (23%)
	Gynecologic	5 (6.7%)
	Prostate	7 (9.3%)
	Other	12 (16%)
Currently receiving chemo	No	15 (20%)
	Yes	60 (80%)
Treatment with curative intent	No	71 (95%)
	Yes	4 (5.3%)

**Table 2:** Treatment Demographics

Variable	Class	No curative intent	Curative intent	Male	Female	Total
Currently receiving chemo	No	15 (100%)		9 (60%)	6 (40%)	15 (20%)
	Yes	56 (93%)	4 (6.7%)	34 (57%)	26 (43%)	60 (80%)

**Table 3:** Cancer Type and Gender

Variable	Class	Male	Female	Total
Cancer type	Breast		11 (34%)	11 (15%)
	Gastrointestinal	15 (35%)	8 (25%)	23 (31%)
	Lung	12 (28%)	5 (16%)	17 (23%)
	Gynecologic		5 (16%)	5 (6.7%)
	Prostate	7 (16%)		7 (9.3%)
	Other	9 (21%)	3 (9.4%)	12 (16%)

The most common types of cancer were lung cancer, colon and rectal cancer. There was an age-related correlation between the gender coupled cancer types in the sample. The female sample afflicted by breast cancer was in the age group 46 - 55, 56 - 65 and 66 - 75 years and male afflicted by colorectal and bowel cancer types was in the age group 56 - 65 years.

### Unmet needs

The ANOVA for SLS by cancer type is presented in Table 4. There was some evidence for between group difference in SLS scores (F = 2.476 with a value = 0.068). Table 5 presents *post hoc* comparisons of lung cancer with breast, colon and other types of cancer showing significant differences with P values of 0.038, 0.029 and 0.022; no other significant differences were observed.

**Table 4:** Comparison of SLS Scores Between Cancer Types Using ANOVA

	Sum of squares	Df	Mean square	F	Sig.
Between groups	1,912.42	3	637.474	2.476	0.068
Within groups	18,279.29	71	257.455		
Total	20,191.71	74			

**Table 5:** *Post Hoc* Dependent Variable: SLS

	(I) Cancer type new	(J) Cancer type new	Mean difference (I-J)	Std. error	Sig.	95% confidence interval	
						Lower bound	Upper bound
LSD		Colon	-0.68548	6.28458	0.913	-13.2166	11.8456
		Lung	-13.11376*	6.20881	0.038	-25.4938	-0.7337
		Other	-1.80008	5.63116	0.750	-13.0283	9.4281
	Colon	Breast	0.68548	6.28458	0.913	-11.8456	13.2166
		Lung	-12.42828*	5.58886	0.029	-23.5721	-1.2844
		Other	-1.11460	4.93922	0.822	-10.9631	8.7339
	Lung	Breast	13.11376*	6.20881	0.038	0.7337	25.4938
		Colon	12.42828*	5.58886	0.029	1.2844	23.5721
		Other	11.31368*	4.84246	0.022	1.6581	20.9693
	Other	Breast	1.80008	5.63116	0.750	-9.4281	13.0283
		Colon	1.11460	4.93922	0.822	-8.7339	10.9631
		Lung	-11.31368*	4.84246	0.022	-20.9693	-1.6581

*The mean difference is significant at the 0.05 level.

Observation of all unmet supportive care needs in the sample has shown that 84% (95% CI: 76-92%) of patients have a moderate or high level of supportive care need on at least one item listed. As shown in Table 6 between 18.7% and 30.7% of patients reported a moderate to high level of need for help on items listed within the domain “symptoms analysis”, the item “dealing with lack of energy or tiredness” being the highest concern. There are 20-24.3% patients reporting a moderate to high level of need on the “psychological” domain as shown in Table 7 with “coping with frustration at not being able to do the things you used to do” being the highest concern. Table 8 presents that 8.1-12% reported a moderate to high level of need on the “activity and daily livings” domain with “getting assistance with preparing meals” being the highest concern. As per Table 9, 9.5-12.2% reported a moderate to high level of need on the “social” domain with “receiving emotional support from friends and family” being the highest concern. Table 10 shows that 9.3-14.9% reported a moderate to high level of need on the “medical information and communication” domain with “getting information about non-conventional treatments” being the highest concern. There are 4.1-11% patients reporting a moderate to high level of need on the “spiritual” domain as presented in Table 11 with “being able to choose the place where you want to die” being the highest concern. Table 12 presents that 9.5-17.3% reported a moderate to high level of need on the “financial” domain with “paying the non-medical costs of your illness (e.g. travel)” being the highest concern.

**Table 6:** Symptoms Analysis

Statement	Percent with moderate to high needs	95% CI
Dealing with lack of energy and tiredness	30.7	20 - 41
Dealing with doing work around the house	25.3	15 - 35
Dealing with changes to your usual routine and lifestyle	22.7	13 - 32
Dealing with a loss of appetite	20.0	11 - 29
Dealing with feeling unwell a lot of the time	18.7	10 - 27

**Table 7:** Psychological

Statement	Percent with moderate to high needs	95% CI
Coping with frustration at not being able to do the things you used to do	24.3	15 - 34
Dealing with concerns about your family’s fears and worries	21.9	12 - 31
Coping with fears about losing your independence	20.3	11 - 29
Working through your feelings about death and dying	20.3	11 - 29
Coping with fears about the cancer spreading	20.0	11 - 29

**Table 8:** Activity and Daily Livings

Statement	Percent with moderate to high needs	95% CI
Getting assistance with preparing meals	12.0	5 - 19
Getting assistance to get up or down stairs	10.8	4 - 18
Dealing with frustration at not being able to drive a car	9.3	3 - 16
Getting assistance with walking	8.1	2 - 14
Getting assistance with showering	8.1	2 - 14

**Table 9:** Social

Statement	Percent with moderate to high needs	95% CI
Receiving emotional support from friends and family	12.2	5 - 20
Being able to express feelings with friends and/or family	10.8	4 - 18
Dealing with maintaining relationships with family members	10.7	4 - 18
Dealing with maintaining relationships with friends	9.5	3 - 16
Coping with talking to family and/or friends about the cancer	9.5	3 - 16

**Table 10:** Medical Information and Communication

Statement	Percent with moderate to high needs	95% CI
Getting information about non-conventional treatments	14.9	7 - 23
Getting information about factors which could influence the course of the cancer	12.2	5 - 20
To be fully informed about your medical test results as soon as possible	10.7	4 - 18
Being able to have open discussion with your doctors	10.7	4 - 18
Getting information about support for yourself, your partner, family or friends	9.3	3 - 16

**Table 11:** Spiritual

Statement	Percent with moderate to high needs	95% CI
Being able to choose the place where you want to die	11.0	4 - 18
Setting new priorities for your life	8.0	2 - 14
Dealing with unmet life goals	5.4	0 - 11
Accepting your relationship with God or a higher being	4.1	0 - 9
Dealing with spiritual issues of death and dying	4.1	0 - 9

**Table 12:** Financial

Statement	Percent with moderate to high needs	95% CI
Paying the non-medical costs of your illness (e.g. travel)	17.3	9 - 26
Dealing with concerns about your financial situation	11.0	4 - 18
Meeting basic living expenses	10.7	4 - 18
Availability of transport to medical appointments, shopping or work	9.7	3 - 17
Obtaining sufficient financial support	9.5	3 - 16

## Discussion

This is the first study conducted in rural Australia that has identified areas in which patients with advanced, incurable cancers have a perceived need for help or assistance. The research has shown that 84% of patients with advanced, incurable cancer have and experience moderate to high needs across a range of domains, which is similar to the work of Rainbird et al [5], where 95% had a perceived need for help. The results show that the existing health care system needs to look into these issues seriously. Areas of unmet needs include both those of the symptoms and psychological needs. This needs a strong re-thinking on the part of care givers who tend to concentrate on the therapeutic aspect of the disease and health side effects following the therapy. Post-therapeutic care thus should include specific nutritional supplementation and physical assistance either as complete physical support or as at least essential assistance. This includes supportive care during washing, brushing, toileting, bathing, dining, walking, taking the prescribed medicines and bedding.

Coordinated cancer care is the cornerstone for advanced cancer patients in rural areas. Providing training in communication skills to clinicians and other healthcare providers can help increase their self-rated confidence in delivering unmet supportive care effectively. In Australia, it is worth mentioning that allied health care staff deliver excellent supportive care to cancer patients. A coordinated workforce can serve as an indispensable solution to deal with these unmet needs filling the gap between individual health care demand and supply. There may also be benefit in the provision of assertiveness training to patients and their care givers to assist them in having needs met.

### Conclusion

This study, the first in rural Australia, has identified areas in supportive care in which the patients with advanced incurable types of cancer have unmet needs. Further, the study has shown that research on developing strategies to deal with cancer related fatigue, and improvements in psycho-social supports, especially in remote areas will serve the purpose of helping advanced cancer patients. The study has also brought to light that there should be greater emphasis to include psycho-social outcome measures in oncology trials and the idea of “personalized cancer therapy” should be broadened encompassing physical, psychological and social aspects of cancer care. Imparting the unmet care needs of patients to the healthcare professionals periodically by training initiatives will enable them to inform the patients on the existing care services available and also give scope for the patients to conceive and communicate their own priorities on supportive care against the backdrop of indefinite treatment outcomes, thus, bridging the care-management gaps in cancer care.
